# Metagenomics next-generation sequencing provides insights into the causative pathogens from critically ill patients with pneumonia and improves treatment strategies

**DOI:** 10.3389/fcimb.2022.1094518

**Published:** 2023-01-13

**Authors:** Ying Liu, Rui Zhang, Bo Yao, Jun Yang, Huimin Ge, Shuyun Zheng, Qi Guo, Jinyan Xing

**Affiliations:** ^1^ Department of Critical Care Medicine, the Affiliated Hospital of Qingdao University, Qingdao, Shandong, China; ^2^ School of Biological Sciences, The University of Hong Kong, Hong Kong, Hong Kong SAR, China

**Keywords:** next-generation sequencing, pneumonia, prognosis, diagnostic effect, ICU

## Abstract

**Background:**

The metagenomics next-generation sequencing (mNGS) is a promising technique for pathogens diagnosis. However, whether the application of mNGS in critically ill patients with pneumonia could cause anti-infection treatment adjustment and thereby affect the prognosis of these patients has not been explored.

**Methods:**

We retrospectively collected the clinical data of patients diagnosed with pulmonary infection in the ICU of the Affiliated Hospital of Qingdao University from January 2018 to January 2021. These patients with pneumonia were divided into mNGS group and no-mNGS group by whether being performed NGS or not. The clinical data, including demographics, illness history, APACHE II score, length of mechanical ventilation, length of stay in the hospital, length of stay in ICU and outcome, were collected. In addition, the data of pathogens and anti-infection treatment before and after NGS were also collected. Propensity score matching was performed to evaluate the mortality and deterioration rate between NGS group and non-NGS group.

**Results:**

A total of 641 patients diagnosed with pneumonia were screened, and 94 patients were excluded based on exclusion criteria. Finally, 547 patients were enrolled, including 160 patients being performed NGS. Among these 160 patients, 142 cases had NGS-positive results. In addition, new pathogens were detected in 132 specimens by NGS, which included 82 cases with virus, 18 cases with fungus, 17 cases with bacteria, 14 cases with mycoplasma, and 1 case with mycobacterium tuberculosis. Anti-infection treatments were adjusted in some patients who performed NGS, including 48 anti-bacterial treatments, 20 antifungal treatments and 20 antiviral treatments. There were no significant differences in the mortality and deterioration rate between NGS and non-NGS group, but it exhibited a trend that the mortality and deterioration rate of NGS group was lower than non-NGS group after the propensity score matching analysis (15.8% vs 24.3%, P=0.173; 25.6% vs 37.8%, P=0.093).

**Conclusion:**

NGS could affect the anti-infection treatments and had a trend of reducing the mortality and deterioration rate of critically ill patients with pneumonia.

## Introduction

Pneumonia, triggered by diverse pathogens like bacteria, virus or fungi, is the most typical infection of patients admitted to the intensive care unit (ICU), with high mortality ranging from approximately 15% to 50% (Li et al., 2016). The source of infection may be one single pathogen, while sometimes it can be accompanied by multiple pathogens. Uncontrolled infection may develop into severe pneumonia, aggravate the inflammation and cause life-threatening organ dysfunction, leading to a poor prognosis (Morris and Medicine, 2018).

Diagnosis and clinical decisions regarding infectious diseases mainly rely on precisely identifying etiologic microorganisms. Bacterial and fungal smear and culture usually work as the gold standard method for identifying causative pathogens. At the same time, polymerase chain reaction (PCR) and antigen tests are currently applied for the detection of viruses. However, traditional pathogen detection methods are time-consuming and inefficient. Empirical antibiotic treatments are often given to patients with negative pathogen results from traditional detection, while the actual pathogens of the infectious source may not be targeted and therefore aggerating the condition of patients (Langelier et al., 2018; Zhang et al., 2021). Early and targeted anti-infection treatments are crucial to reduce pneumonia mortality rate (Xie et al., 2019).

With the rapid development of molecular methods, metagenomic sequencing, also called next-generation sequencing (NGS), emerged as a fast and precise diagnosis of the infection (Behjati and Tarpey, 2013). Compared to the traditional microorganism culture, NGS directly sequence all the nucleic acid fragments in a short time with high-throughput capacity (Gwinn et al., 2019). The results of bioinformatics analyses will precisely illustrate the species of the pathogen within the sample, especially for the rare and slow-growing microorganisms or multiple infections, leading to fast and accurate diagnosis and treatment.

In recent years, the value of NGS has gradually been recognized for infection diagnosis owing to the high efficiency and high positive detection rate in culture-negative samples (Brenner et al., 2018; Tarabichi et al., 2018). However, whether the NGS results can affect medical decisions and consequently improve the prognosis of patients with pneumonia in the ICU has not been reported. The objective of this study is to evaluate the value of NGS in pulmonary infection diagnosis and pathogen identification in ICU patients, by comparing the result of NGS to the conventional detection methods.

## Methods

### Study design

A retrospective screen was performed from January 2018 to January 2021 for cases of pulmonary infections and pneumonia admitted into the ICU of The Affiliated Hospital of Qingdao University. Patients that had been discharged from hospital before we obtained NGS results were excluded. The specimens of NGS results included blood, bronchoalveolar lavage fluid or sputum. This study was conducted according to the Declaration of Helsinki principles and approved by the Ethics Review Committee of The Affiliated Hospital of Qingdao University (Approval number: QYFYWZLL26515), and was registered on the Chinese Clinical Trial Registry (Registration number: ChiCTR2100050201).

The individual informed consent was waived for this retrospective analysis.

### Clinical data collection and antibiotic treatment

Data of targeted patients were initially screened from the registration of discharge and admission, and further information was collected from the medical records through the Hospital Information System (HIS), including demographics, laboratory test results, APACHE II score, length of mechanical ventilation, length of stay in hospital and ICU, initial antibiotics at ICU admission and adjustment later based on the pathogen results of NGS were also collected. These patients with pneumonia were divided into the NGS group and the non-NGS group by whether they were being performed NGS or not.

### Statistical analysis

The t-test was used to determine the normal distribution and uniformity of variance. Also, the student t-test and χ2 test were used to calculate differences in continuous variables between groups. Propensity score matching (PSM) was performed to more precisely evaluate the diagnostic effects of NGS. Data analyses were performed using the SPSS 26.0 (IBM, Armonk, NY, USA) software. P values < 0.05 were considered significant.

## Results

### Participants and study design (sample and patient characteristics)

**Figure 1:**
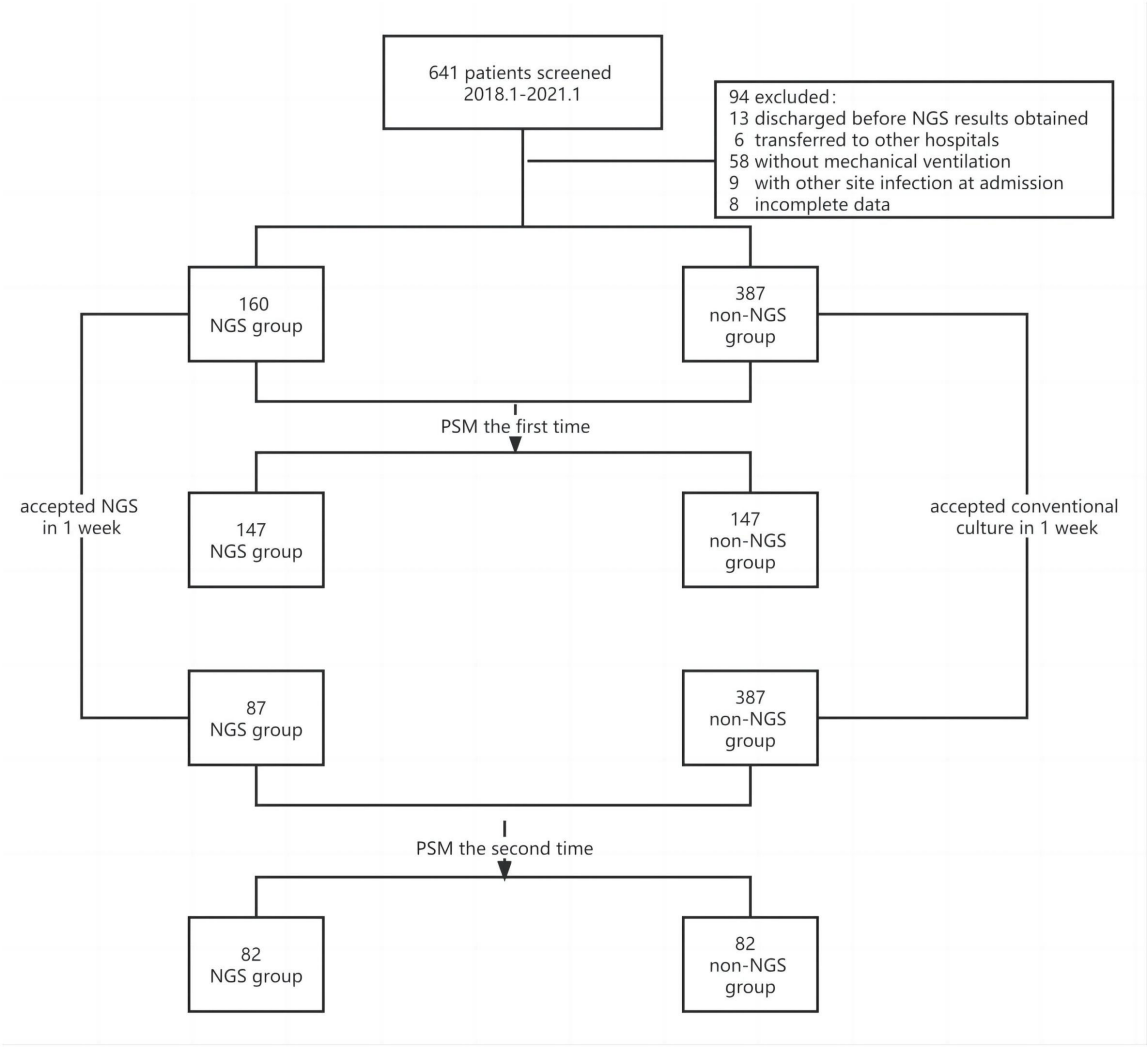
Flow chart of patients included.

The flow chart of patients included was shown in **Figure 1**. A total of 641 patients were screened, and 94 patients were excluded based on exclusion criteria. 547 (85.3%) patients were enrolled, among which 160 (29.3%) patients got NGS detection, and 387 (70.7%) patients had conventional detection. The demographic and baseline characteristic of patients enrolled were provided in **Table 1**. Neither group had significant differences in gender, age and medical history (P >0.01). The APACHE II scores and diabetes ratio were higher in patients who performed NGS (P<0.001). To adjust the imbalanced distribution, propensity score matching (PSM) was performed to more precisely evaluate the diagnostic effects of NGS and conventional methods. NGS (n = 160) patients were 1:1 propensity-matched to non-NGS (n = 387) based on the APACHE II Score.

**Table 1:** Baseline characteristics and clinical indices of NGS and non-NGS groups.

	NGS (n=160)	non-NGS (n=387)	P value
Gender (male/female)	114/46	236/151	0.023
Age (years)	63 (53-73)	65 (52-75)	0.282
APACHE II Score	22 (17-26)	17 (12-23)	<0.01
Cardiovascular disease (N/Y)	88/72	176/211	0.043
Cerebrovascular disease (N/Y)	135/25	331/56	0.729
Diabetes (N/Y)	107/53	309/78	0.001
COPD (N/Y)	159/1	372/15	0.048
Cancer (N/Y)	143/17	357/30	0.275
Autoimmune disease (N/Y)	154/6	364/23	0.298
Etiological tests results times(hours)	56.91 ± 8.82	74.96 ± 15.81	<0.01
Duration of mechanical ventilation (d)	13 (6-28)	4 (0-12)	<0.01
Length of stay in ICU (d)	21 (13-42)	12 (5-23)	<0.01
Length of stay in hospital (d)	29 (17-50)	19 (10-35)	<0.01
Mortality rate (%)	20% (32/160)	18.1% (70/387)	0.601
Deterioration rate (%)	33.1% (53/160)	28.7% (111/387)	0.302

### Clinical impact of NGS-based diagnostics

**Figure 2:**
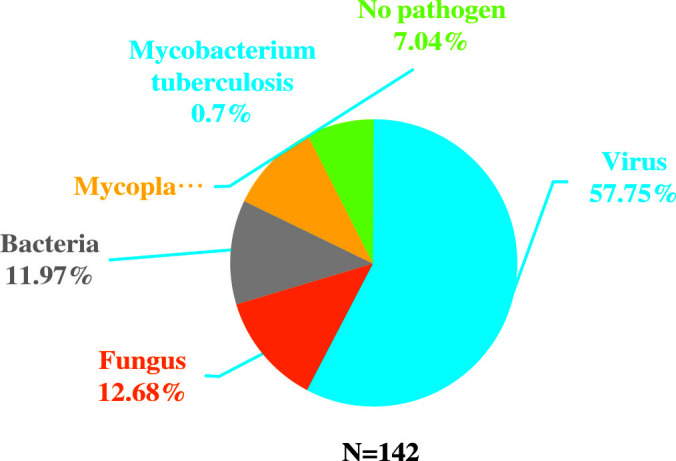
Extra pathogens detected by NGS compared to conventional detection methods.

Among all the patients with pulmonary infections, the percentage of NGS-positive results was 88.75% (142/160), while the positive ratio of conventional methods was 86.25% (138/160). There were no significant differences in the detection rate. However, compared to conventional methods, NGS was more sensitive in detecting pathogens (**Figure 2**). Among the 142 NGS-positive cases of pulmonary infections, extra pathogens were seen in 132 specimens, including 82 cases with viruses, 18 with fungus, 17 with bacteria, 14 with mycoplasma, and one with *mycobacterium tuberculosis.*

### Identification of pathogens in negative conventional pathogen detection specimens by NGS

**Figure 3:**
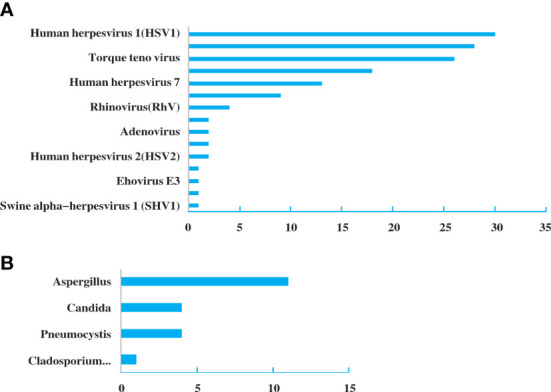
Pathogen spectrum of specimens in the NGS group. **(A)**. Bacterial spectrum. **(B)**. Fungal spectrum.

A total of 31 specimens received negative or non-specific results from conventional pathogen detection tests, in which 23 had positive results and 8 had negative results by NGS. Among the specimens of pulmonary infectious patients enrolled in this study, the substantial pathogens that traditional tests failed to detect included 18 specimens with bacterial, 14 with viruses, six with fungus and two with mycoplasma and *pneumocystis carinii* (**Figure 3**).

### Antibiotic adjustment affected by the diagnostic effect of NGS

**Figure 4:**
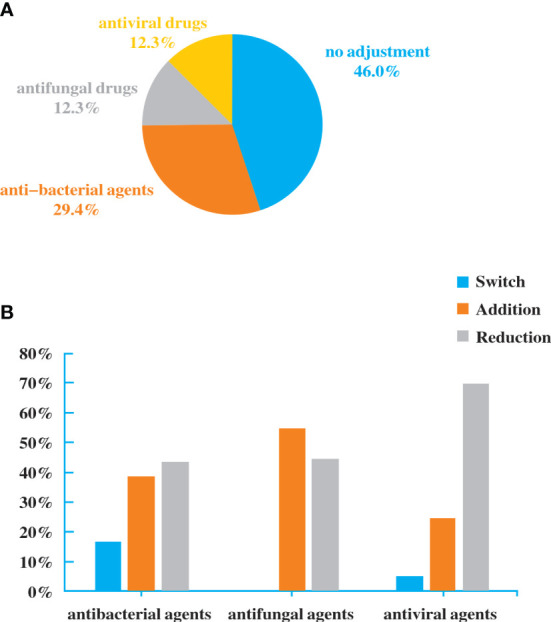
Antibiotic adjustment affected by the diagnostic result of NGS. **(A)**. Bacterial spectrum. **(B)**. Fungal spectrum.

Antibiotics were adjusted in patients who performed NGS. Among the 160 patients, 48 (30%) had added or stopped anti-bacterial agents, 20 (12.5%) adjusted antifungal drugs, and 20 (12.5%) adjusted antiviral drugs (**Figure 4A**). The reduction rate was higher than addition rate in anti-bacterial and antiviral agents (**Figure 4B**). Although there was no significant difference, it exhibited a trend of purposefully reducing complete pathogen covered empirical antibiotics.

### Effect of NGS compared with traditional detection methods

After the 1:1 propensity matching, NGS (n = 147) and non-NGS (n = 147) groups were adequately balanced, and 147 cases were included for further analysis (**Table 2**).

**Table 2:** Baseline characteristics and clinical indices of NGS and non-NGS groups after PSM balancing.

	NGS (n=147)	non-NGS (n=147)	P Value
Gender (male/female)	103/44	98/49	0.531
Age (years)	63 (53-73)	65 (51-75)	0.355
APACHE II Score	22 (16-25)	20 (15-26)	0.643
Cardiovascular disease (N/Y)	80/67	83/64	0.725
Cerebrovascular disease (N/Y)	127/20	128/10	0.863
Diabetes (N/Y)	103/44	98/49	0.531
COPD (N/Y)	146/1	147/0	0.9999
Cancer (N/Y)	133/14	135/12	0.681
Autoimmune disease (N/Y)	142/5	142/5	1
Etiological tests results times(hours)	57.10 ± 9.01	77.01 ± 16.98	<0.01
Duration of mechanical ventilation (d)	12 (7-28)	5 (1-15)	<0.01
Length of stay in ICU (d)	21 (12-42)	12 (5-25)	<0.01
Length of stay in hospital (d)	29 (17-50)	19 (10-34)	<0.01
Mortality rate (%)	21.1% (31/147)	25.9% (38/147)	0.335
Deterioration rate (%)	33.3% (49/147)	37.4% (55/147)	0.464

However, some of the patients were performed NGS after an extended stay in the ICU. The time we performed NGS detection was not the actual time of admission to the ICU, the results can be affected by this confounding factor. Consequently, we screened the patients under NGS detection within a week and then performed a matching analysis of 87 cases, 82 cases were matched and included in the final analysis. The baseline analysis is provided in **Table 3**, and the matching results are provided in **Table 4**. There was no significance between NGS and non-NGS groups in mortality rate (P=0.173) and deteriorated rate (P=0.093). Compared to Non-NGS patients, the length of stay in ICU and hospital and the duration of mechanical ventilation was longer in NGS patients than in non-NGS patients.

**Table 3:** Baseline characteristics and clinical indices of NGS and non-NGS groups of 87 cases.

	NGS (n=87)	non-NGS (n=387)	P Value
Gender (male/female)	60/27	236/151	0.165
Age (years)	60 (52-72)	65 (52-75)	0.080
APACHE II Score	22 (17-26)	17 (12-23)	<0.01
Cardiovascular disease (N/Y)	47/40	176/211	0.149
Cerebrovascular disease (N/Y)	77/10	331/56	0.469
Diabetes (N/Y)	59/28	309/78	0.015
COPD (N/Y)	87/0	372/15	0.085
Cancer (N/Y)	75/12	357/30	0.073
Autoimmune disease (N/Y)	84/3	364/23	0.445
Etiological tests results times(hours)	57.63 ± 9.22	74.96 ± 15.81	<0.01
Duration of mechanical ventilation (d)	10 (6-21)	4 (0-12)	<0.01
Length of stay in ICU (d)	27 (10-32)	12 (5-23)	<0.01
Length of stay in hospital (d)	21 (12-39)	19 (10-35)	0.124
Mortality rate (%)	17.2% (15/87)	18.1% (70/387)	0.852
Deterioration rate (%)	26.4% (23/87)	28.7% (111/387)	0.674

**Table 4:** Characteristics and clinical indices of NGS and non-NGS groups after propensity matching.

	NGS (n=82)	Non-NGS (n=82)	P Value
Gender (male/female)	58/24	54/28	0.502
Age (years)	60 (53-72)	62 (47-72)	0.853
APACHE II Score	22 (17-26)	20 (14-27)	0.356
Cardiovascular disease (N/Y)	44/38	51/31	0.268
Cerebrovascular disease (N/Y)	72/10	78/4	0.094
Diabetes (N/Y)	57/25	59/23	0.731
COPD (N/Y)	82/0	82/0	1
Cancer (N/Y)	73/9	73/9	1
Autoimmune disease (N/Y)	79/3	80/2	0.999
Etiological tests results times(hours)	57.80 ± 8.67	76.05 ± 17.55	
Duration of mechanical ventilation (d)	10 (6-21)	5 (1-14)	0.002
Length of stay in ICU (d)	17 (10-33)	11 (4-23)	0.01
Length of stay in hospital (d)	22 (12-39)	18 (8-31)	0.041
Mortality rate (%)	15.8% (13/82)	24.3% (20/82)	0.173
Deterioration rate (%)	25.6% (21/82)	37.8% (31/82)	0.093

**Figure 5:**
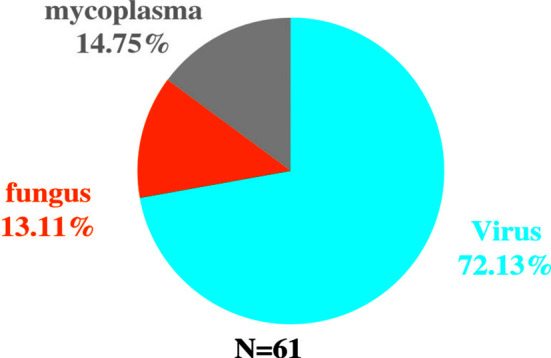
Extra pathogens detected by NGS compared to conventional detection methods after propensity matching.

**Figure 6:**
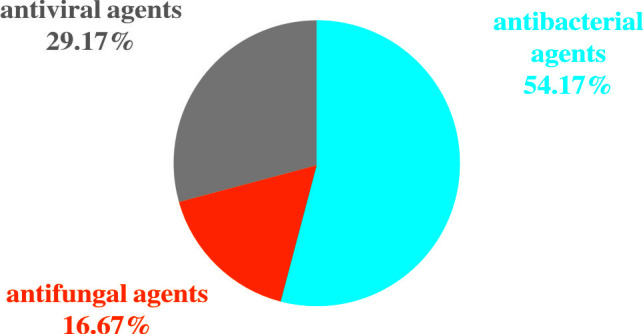
Antibiotic Adjustment affected by the diagnosis of NGS after propensity matching.

Among the 82 NGS-positive cases after propensity matching, extra pathogens were seen in 61 specimens, including 44 (72.13%) cases with viruses, 9(14.75%) with mycoplasma and 8 (13.11%) with fungus (**Figure 5**).48 of the patients had their antibiotics adjusted. 26 (54.17%)had adjusted anti-bacterial agents,14 (29.17%) adjusted antiviral drugs and 8 (29.17%)adjusted antifungal drugs (**Figure 6**).

## Discussion

Our retrospective study compared the diagnostic effects of NGS with traditional detection methods, evaluating its impact on antibiotic adjustment and the prognosis of pneumonia patients in the ICU. NGS exhibited fast and comprehensive detection efficacy for mixed pathogens and may potentially prompt more precise antibiotic treatment, thus improving the prognosis of patients with pulmonary infections.

Pneumonia, a commonly occurred respiratory infection, is responsible for approximately 30% of mortality in the patients admitted to ICU (Walden et al., 2014; Rider and Frazee, 2018). While widely being described as bacterial pneumonia, it has spread out all around the world in the recent pandemic with the novel coronavirus (COVID-19), whose mortality rate accounts for 35-50% (Richardson et al., 2020). Pneumonia caused by mixed pathogens is still a significant concern in the ICU. Precise and timely detection of pathogens can optimize antibiotic administration, potentially reverse the progress of the disease, shorten the length of stays and decrease the mortality rate. However, with low concentration levels of the pathogen-associated substances, pathogen detection in the early stage of infection can be non-specific and inefficient, resulting in delayed diagnosis, impeding targeted therapies or causing excessive antibiotic treatment, and eventually leading to poor prognosis (Shen et al., 2020; Zhang et al., 2021). Also, distinguishing some infectious diseases from non-infectious diseases can be really challenging relying only on routine laboratory tests and imaging analysis. Considering patients in the ICU generally combine several underlying diseases, it is more critical to improve the diagnostic efficiency and perform precise treatment (Huang et al., 2020).

Although accurate diagnosis of pulmonary infection is crucial to the improved management and better prognosis, how to precisely detect and identify pathogens remains a big challenge. Conventionally, diagnosis of infection mainly relies on serological analysis, molecular techniques, histopathology, and smear microscopy (Das et al., 2018; Qian et al., 2020). However, conventional diagnostic practices, like microbial cultures and polymerase chain reaction (PCR), are commonly time-consuming, labor-intensive, and usually lack adequate sensitivity and selectivity (Kumar et al., 2019).

Some precise detection methods have emerged with the urgent need of diagnostic technology, among which the NGS exhibits potential for improving clinical practice and public health through high-throughput technologies and bioinformatics (Gwinn et al., 2017; Gwinn et al., 2019). Our results showed that the percentage with NGS-positive results was 88.75% (142/160). Although there was no significant difference compared to traditional method (86.25%), NGS presented high sensitivity for identifying specific pathogens that are usually not detected in conventional tests. Among the 142 NGS-positive cases of pulmonary infections, 132 specimens had extra pathogens detected, of which 82 cases were virus. *Human herpesvirus* and *Torque teno virus* were the most common pathogens. This might be related to the detection of latent viruses, or it is originated from the reactivation of latent herpesviruses. Studies showed that HSV reactivation is common in ICU patients and associated with increased morbidity and mortality (Luyt et al., 2020). In addition, HSV reactivation is the initial step toward HSV-associated bronchopneumonitis, which can aggravate lung inflammation or damage and facilitate the occurrence of nosocomial bacterial pneumonia, resulting in prolonged mechanical ventilation. It is an alert that attention should be paid to specific virus infections in critically ill patients with pneumonia. Besides, NGS diagnosis may potentially affect clinical decision. In our study, 48 (30%) patients had anti-bacterial agents added or stopped, 20 had antifungal drugs adjusted and 20 had antiviral drugs adjusted based on the NGS results. The NGS results may contribute to more comprehensively evaluating the empiric antimicrobial therapy and making effective adjustments for critically ill patients with pneumonia in the ICU. The reduction of antibiotics can avert excessive antibiotic treatment and unnecessary healthcare costs while the addition of drugs may target specific pathogens. The mortality and deterioration rate were further analyzed after the 1:1 propensity matching, and a significant difference in APACHE II Score was displayed between the two groups. Considering the time of the admission to the ICU and NGS tests was not the same, we screened the patients that were accepted NGS after being admitted to the ICU within a week, and then completed a matching analysis of 87 cases. 82 cases were matched and included in the final analysis. Although there was still no significant difference between NGS and non-NGS groups in mortality rate (P=0.173) and deterioration rate (P=0.093), it presented a declining trend in the NGS group. We then shortened the difference of time to two days; the matching cases were too few for further analysis. If we enlarge sample size, the two groups’ mortality and deterioration rates may present significant differences. Compared to non-NGS patients, the length of stay in ICU and hospital and the duration of mechanical ventilation was longer in NGS patients. The total hospitalization expenses in NGS patients were significantly higher than in non-NGS patients. It is probably because in most ICUs in China, except for specific clinical trials, only when the conditions of patients got worse or the empirical treatment did not work well, the application of NGS were then considered. That is to say, bias obviously existed in the choice of NGS diagnosis, and it may severely affect the results.

In this study, we retrospectively compared the subsequent effects of NGS detection on the prognosis of patients with pneumonia in the ICU and performed propensity score matching based on APACHE II score. The innovative strength of our study is that, by far this is the largest amount of cases analysis on comparing antibiotic management and the prognosis of critically ill patients with pneumonia in the ICU. The clinical data, including demographics, illness history, APACHE II score, length of mechanical ventilation, length of stay in the hospital, length of stay in ICU, and prognosis, were collected and analyzed. For a more precise analysis of prognosis, we applied propensity score matching to reduce the bias due to confounding variables that the severity of patients’ condition may cause. Similarly, in recent articles, Xie et al., reported that the 28-day mortality of the NGS group was significantly lower than the control group (16.7% vs 37.7%, p = 0.008) (Xie et al., 2019). In another study, Zhang. et al. came to similar conclusion that the 28-day mortality rate of the NGS group was significantly lower than that of the non-NGS group (21.4% vs 49.1%, P = 0.006) (Zhang et al., 2020). Consistent with our results, the mortality rate (15.8%) and deterioration rate (24.3%) in NGS group were both lower than in non-NGS group at 25.6% and 37.8% respectively. Although there was no significant difference between the two groups in mortality rate (P = 0.335 to 0.173) and deterioration rate (P = 0.464 to 0.093), it showed the trend that the rate of the NGS group tend to be lower than that in the non-NGS group as we gradually reduced the disturbance of the time length between admission and NGS detection. The NGS detection presented the potential to improve the prognosis of patients with pneumonia.

Our results showed that NGS produced results much faster than traditional methods by about 24 hours or more.At present, our specimens still need to be sent to other places for testing, because the local laboratory is still under construction. Furthermore,NGS is not currently covered by medical insurance in our country. The above resulted in bias in patient selection in this study. If NGS could be tested in hospitals, or if it was cheaper, its advantages might be even greater.

The advantages made NGS a novel, fast, and precise method to better diagnose pulmonary and any other infections of patients. However, conventional detection methods like blood culture are still our first choice. The reasons why NGS has not been served as a routine clinical diagnostic method are quite obvious. Significant barriers out there hinder the further application of NGS in the routine diagnosis of infection: to obtain precise and fast analysis, sophisticated bioinformatics systems, fast data processing and extensive data storage capabilities are required, which drives up the cost. Besides the equipment cost, professional researchers are also highly needed to analyze and clinically interpret the data (Simner et al., 2018). Therefore, it has not been a routine test for the clinic to diagnose infectious diseases and consequently, there is no universal criteria and authoritative guidelines to interpret the report (Zhang et al., 2020).

First, as a retrospective study, the current situation of the NGS application in our hospital mentioned above led to bias in the severity of patients. The application of NGS can even be considered as a process to screen patients ahead of this study. There is no doubt that the condition of patients with NGS detection was worse than that of the non-NGS group, which was precisely reflected in the significant difference between the NGS group and the non-NGS group in the APACHE II score. Second, the time that NGS diagnosis was performed varied a lot. Although we used propensity score matching to reduce the disturbance, the results were still possibly affected by this factor. Also, patients with ventilation and extracorporeal membrane oxygenation (ECMO) were included for analysis. However, the types of pulmonary infections were not specifically distinguished. Finally, our study was also limited by the feature of a single-centred retrospective study. A prospective and multicenter randomized controlled trial is required to comprehensively analyze the essential role of NGS in the whole process of clinical decision-making, including diagnosis, treatment and more importantly, the evaluation of the prognosis of patients.

## Conclusion

Our study showed that the accurate and fast detection efficacy enabled NGS to potentially prompt precise antibiotic treatment, improving the prognosis of patients with pulmonary infection in the ICU. The NGS may be a potential substitutable method for the diagnosis of pulmonary infection. However, the complex requirements to perform NGS stalled its application in becoming a routine clinical diagnostic method. For now, it can only be used to assist the clinical diagnosis.

## Data availability statement

The raw data supporting the conclusions of this article will be made available by the authors, without undue reservation.

## Ethics statement

The studies involving human participants were reviewed and approved by the Ethics Review Committee of The Affiliated Hospital of Qingdao University. Written informed consent for participation was not required for this study in accordance with the national legislation and the institutional requirements.

## Author contributions

All authors contributed to the study conception and design. JX contributed to the conception and design of the research; YL and QG collected data and drafted the manuscript; RZ contributed to the design of the research; BY contributed to the literature search and review of manuscript; HG and SZ contributed to the acquisition and analysis of the data. All authors commented on previous versions of the manuscript. All authors contributed to the article and approved the submitted version.
